# Chronic Jet Lag Simulation Decreases Hippocampal Neurogenesis and Enhances Depressive Behaviors and Cognitive Deficits in Adult Male Rats

**DOI:** 10.3389/fnbeh.2019.00272

**Published:** 2020-01-08

**Authors:** Emily A. Horsey, Teresa Maletta, Holly Turner, Chantel Cole, Hugo Lehmann, Neil M. Fournier

**Affiliations:** Department of Psychology, Trent University, Peterborough, ON, Canada

**Keywords:** circadian disruption, depression, anhedonia, learning and memory, anxiety, emotionality, neurogenesis, hippocampus

## Abstract

There is a long history that protracted periods of circadian disruption, such as through frequent transmeridian travel or rotating shift work, can have a significant impact on brain function and health. In addition, several studies have shown that chronic periods of circadian misalignment can be a significant risk factor for the development of depression and anxiety in some individuals with a history of psychiatric illness. In animal models, circadian disruption can be introduced through either phase advances or delays in the light–dark cycle. However, the impact of chronic phase shifts on affective behavior in rats has not been well-studied. In the present study, male rats were subjected to either weekly 6 h phase advances (e.g., traveling eastbound from New York to Paris) or 6 h phase delays (e.g., traveling westbound from New York to Hawaii) in their light/dark cycle for 8 weeks. The effect of chronic phase shifts was then examined on a range of emotional and cognitive behaviors. We found that rats exposed to frequent phase advances, which mirror conditions of chronic jet lag in humans, exhibited impairments in object recognition memory and showed signature symptoms of depression, including anhedonia, increased anxiety behavior, and higher levels of immobility in the forced swim test. In addition, rats housed on the phase advance schedule also had lower levels of hippocampal neurogenesis and immature neurons showed reduced dendritic complexity compared to controls. These behavioral and neurogenic changes were direction-specific and were not observed after frequent phase delays. Taken together, these findings support the view that circadian disruption through chronic jet lag exposure can suppress hippocampal neurogenesis, which can have a significant impact on memory and mood-related behaviors.

## Introduction

Proper circadian rhythms are an important feature of normal health enabling organisms to adapt to daily changes in their environment. Frequent transmeridian travel and rotating shift work are well-known disruptors of this internal timing system in humans (Waterhouse et al., 2007). In some individuals, short-term misalignment between the endogenous circadian clock and the desired destination sleep/wake schedule can produce a temporary “*jet lag*” disorder, which is associated with symptoms of fatigue, gastrointestinal distress, reduced psychomotor coordination, and diminished cognition and mood. These symptoms generally dissipate as the circadian clock gradually entrains to the new destinations’ time. However, with sustained periods of circadian disruption, a host of adverse health outcomes and clinical pathologies can occur, including a higher incidence of cancer, diabetes, obesity, cardiovascular diseases, and early mortality (Ekstrand et al., 1996; Maywood et al., 2006; Greene, 2012; Mota et al., 2017; Gotlieb et al., 2018; Lin and Farkas, 2018).

Long-term, repeated disturbances of the internal circadian system has been documented to adversely impact the brain (for review, see, Hastings et al., 2003). For example, flight attendants who had experienced repeated jet lag with limited recovery period between flights were found to have reduced temporal lobe volume and exhibited spatial learning deficits when compared to a ground crew control group (Cho, 2001). Interestingly, these cognitive deficits were long lasting—extending for several years—and were associated with elevated levels of the stress hormone cortisol (Cho et al., 2000), a key component of the hypothalamic-pituitary-adrenal stress axis (Sapolsky, 2015). In addition, chronic sleep disturbances have been associated with reduced cortical gray matter volume in several brain regions, including the prefrontal cortex (Altena et al., 2010; Sexton et al., 2014) and hippocampus (Joo et al., 2014). These findings underscore that structural and functional brain adaptations can be downstream effects associated with frequent episodes of circadian dysrhythmia and sleep loss.

Experimental conditions of jet lag can be simulated in rodents through either advancing or delaying the onset of when housing lights are turned off. Using this approach, there is clear evidence that repeated phase shifts of the light/dark (LD) cycle can produce disruptions to learning and memory processes in rats with phase advances typically inducing the greatest impairment (Tapp and Holloway, 1981; Fekete et al., 1985; Devan et al., 2001; Craig and Mcdonald, 2008; Loh et al., 2010; Phan et al., 2011). While the adverse effect of chronic circadian disruption on cognition in rodents and humans is clear, the impact of these changes on affective behaviors is less known. Nonetheless, there is a long history demonstrating that repeated periods of circadian misalignment can contribute to the etiology of mood and anxiety disorders (Vadnie and Mcclung, 2017). For instance, jet lag was found to be a significant precipitator of depressive symptoms in some individuals with a history of psychiatric illness (Tec, 1981; Jauhar and Weller, 1982; Young, 1995; Katz et al., 2002). In rodents, disruption of the natural circadian rhythm by constant lighting increases anxiety-like and depressive-like behavior (Tapia-Osorio et al., 2013). Furthermore, mice with mutations of circadian clock genes show abnormal emotional behaviors (Roybal et al., 2007; Kovanen et al., 2013; Landgraf et al., 2016a). These findings suggest that the proper entrainment of circadian rhythms is necessary for normal functioning of neural circuits that control emotion and regulate stress reactivity (Bedrosian and Nelson, 2017).

Despite the increasing awareness of the health risks associated with chronic jet lag, the neurobiological factors that underlie the accompanied changes in mood and cognitive behavior remain poorly understood. To address this, we exposed adult male rats to 8-weeks of frequent (6-h) phase delays or phase advances to the LD cycle to investigate the impact of chronic circadian disruption on behavioral measures related to cognitive and affective behavior. This procedure has been used extensively to simulate conditions of experimental “jet lag” in rodents (Filipski et al., 2004; Davidson et al., 2006; Craig and Mcdonald, 2008; Gibson et al., 2010; Kott et al., 2012). In addition, we examined whether chronic jet lag conditions could alter levels of hippocampal neurogenesis. Our findings demonstrate that chronic phase advancements of the LD cycle produce clear impairments in object recognition memory along with increases emotional responses, and that disruption in neurogenic processes in the hippocampus may contribute to the changes in affective and cognitive behavior associated with chronic jet lag conditions.

## Materials and Methods

Male Sprague Dawley rats, weighing between 200 and 220 g, were purchased from Charles River Laboratories (Montreal, QC, Canada). Upon arrival, rats were acclimated to the housing facility and were single housed in standard polypropylene cages with corn bedding. Rats were given free access to food and water for the duration of the study unless otherwise noted. Ambient temperature in the housing rooms was maintained between 21 ± 1°C. All experimental procedures were approved by Trent University Animal Care Committee and were in accordance with the Canadian Council on Animal Care (CCAC). Efforts were made to minimize the number of experimental animals used.

### Experimental Groups

**FIGURE 1:**
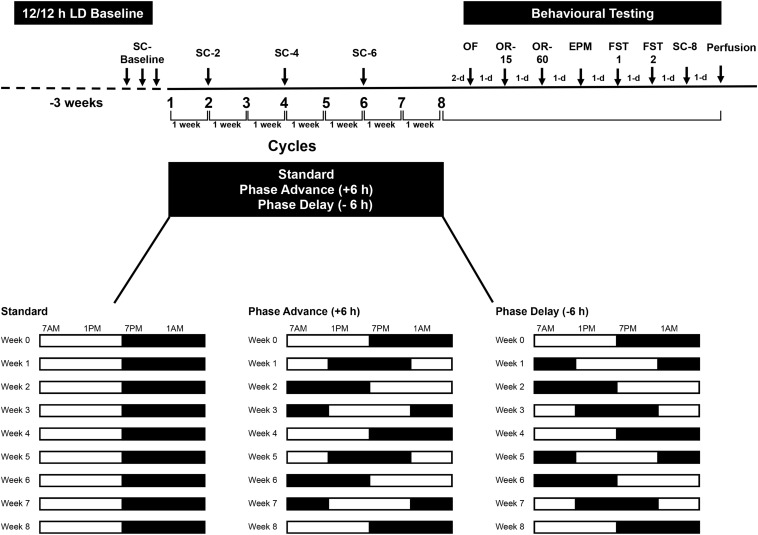
Experimental design. Rats were housed on a standard 12/12 LD cycle for 3 weeks before the start of experiment. Rats were subjected to chronic phase advance (*n* = 8) or delay (*n* = 8) protocols, which consisted of weekly 6 h phase advances or 6 h delays of the LD cycle. A group of rats (*n* = 8) that remained on the standard LD cycle served as controls. All shifts took place on Monday. For 3 days prior to the first phase shift, rats were habituated to a sucrose solution (baseline sucrose consumption). Two days after the 8^th^ final shift, rats underwent behavioral testing: open field (OF), object recognition test with 15 min retention interval (OR-15), object recognition test with 60 min retention interval (OR-60), elevated plus maze (EPM), and 2 days of forced swim test (FST1 and FST2). Rats were euthanized the day after the last sucrose consumption test. Sucrose consumption tests were conduct at the end of shifts 2, 4, 6 and at the end of behavioral testing (shift 8).

All rats were initially maintained on a standard 12:12 h LD cycle with lights on at 0700 h and off at 1900 h for 3-weeks prior to the start of the experiment. During the study, luminance was provided by a fluorescent white light (∼200–300 lux at cage level). Following acclimation to these conditions, the rats were assigned randomly to either remain under the standard LD cycle (*n* = 8) or placed on a chronic LD shift schedule (*n* = 8 per group for each of the lighting regimens, *see below*) to simulate the conditions of repeated jet lag. The procedures were carefully controlled as to match those used in a previous study (see Kott et al., 2012). The shifting protocols consisted of either shortening the light period by 6 h (phase advance – similar to the effects of eastward travel from New York To Paris) or lengthening the dark period by 6-h (phase delay – similar to the effects of westward travel from New York to Hawaii) for 8-weeks (Figure 1). After each 6 h phase advance or delay, the rats were left undistributed to allow adjustment until the next phase shift, which occurred 7 days later. This resulted in 8 weekly LD shifts over the course of the study. Beginning after the 8^th^ phase advance/-delay LD shift, all rats were assessed on a battery of tests [open field test, elevated plus maze, forced swim tests (FSTs), and object recognition tests]. The open field test, elevated plus maze, and FSTs were used to examine changes in emotional behavior, such as anxiety and depressive-like behavior, whereas the object recognition test was used to examine learning and short-term memory. Throughout the experiment, sucrose consumption was used to assess for anhedonia, at phase shifts 2, 4, 6, and at the completion of behavior testing.

### Behavioral Testing

All behavioral experiments began 48 h after the final LD cycle shift (8^th^ cycle) and were performed during the light phase (1000 and 1600 h). The order of behavioral testing was as follows: open field test (2 habituation sessions), object recognition test 1 (retention interval: 15 min), object recognition test 2 (retention interval: 60 min), elevated plus maze, and 2 days of FST. As discussed above, sucrose consumption tests (see below) were conducted at multiple times during the experiment and on the day after the FST (please see Figure 1 for timeline of behavioral testing).

### Sucrose Consumption Tests

Anhedonic-like behavior was evaluated by monitoring of sucrose intake using a single bottle test. Rats were habituated to a 1.5% sucrose solution for 3 days prior to the first LD cycle shift. This allowed for the estimation of baseline sucrose consumption before beginning the phase advance or delay of the LD cycles. Sucrose consumption was measured after the 2^nd^, 4^th^, and 6^th^ LD shifts, as well as after behavioral testing (i.e., end of 8^th^ LD cycle). For the test, the rats were deprived of food and water overnight. Following overnight fluid and food deprivation, the rats were exposed to a pre-weighed 1.5% sucrose solution bottle for 1 h. The total amount of sucrose water consumed during the 1 h test was evaluated at the end of cycles 2, 4, and 6. Food and water was immediately resumed after testing before initiating the next cycle shift. As a control, water intake was measured over a 1 h and 24 h period the day after completing a sucrose consumption test. Sucrose consumption was estimated by calculating the ratio of sucrose water consumed over tap water consumed multiplied by 100.

After completion of behavioral testing (cycle 8), a final sucrose consumption test was completed. The rats underwent a period of overnight fluid and food deprivation as described above. Following this period, the rats were exposed to a preweighed 2.0% sucrose solution bottle for 24 h. The total amount of sucrose consumption during this period was measured.

### Open Field Test

Two days after the initiating the final LD cycle shift (8^th^ shift), the rats from all three groups were given two separate 10 min exposures to freely explore a novel open field arena. The arena was a stainless steel 60 cm × 60 cm × 60 cm open square box. Each arena contained a small amount of corn bedding, fully covering the flooring. During the exploration period, the distance traveled, and the time spent in the peripheral (46.6 cm × 46.6 cm) and central (24 cm × 24 cm) compartments of the arena well as the movement velocity was recorded by a video camera mounted directly above the apparatus. All behaviors were analyzed using Any-Maze software (v. 4.71, Stoelting, Co., United States). Immediately after exploration, the rat was returned to its home cage and was left undisturbed until the next exploration session. The inter-session interval was approximately 6 h. To minimize the impact of olfactory cues, the chambers were cleaned with Oxivir Five 16 concentrate (Diversey, Inc., Canada) prior to testing each rat.

### Object Recognition Tests

Novel object recognition testing was carried in the same empty open field arena as described above and was completed the day after open field testing. The test consisted of two phases: a sample trial and a retention (test) phase. For the sample phase, each rat was allowed 5 min to freely explore the arena, which now contained two identical objects. The object pairs were located 15 cm from each adjacent wall. A retention test was conducted 15 min later. During this test, one of the objects previously presented was replaced by a new object. The rats were returned to the open field arena and allowed to explore for 5 min during the test phase. Object investigation was defined as sniffing (within ∼3 cm) or touching the object with the nose and/or forepaws. Turning around or sitting on the object was not considered exploratory behavior. The time spent by the rats investigating each object, familiar or novel, during the test phase was recorded by a video camera and was scored by a researcher blind to the housing condition of each subject. All object pairs as well as the position of the novel object during the test were counter-balanced across rats. A discrimination index (DI) was calculated as follows: DI = (novel object)/(novel object + familiar object) multiplied by 100, this ratio represents the time spent investigating the novel object expressed as a proportion of the total time spent investigating both objects. The open field arena was cleaned with 70% alcohol and air-dried prior to the commencement of each trial for every rat.

Twenty-four hours later, each rat was placed in the same open field arena as before, but the arena now contained a new set of objects. The rat was allowed to explore the arena and the object pairs for 5 min. A retention test was carried out 1 h later, and one of the objects presented previously was replaced by a novel object. As before, the time spent by investigating the novel and familiar objects was recorded and a discrimination index was calculated.

### Elevated Plus Maze

Anxiety-like behavior was assessed using an elevated plus maze consisting of two open arms (50 cm × 10 cm) and two enclosed arms (50 cm × 10 cm × 40 cm), elevated 50 cm from the floor. The plus maze was placed in the center of a homogeneously illuminated room. Each rat was placed in intersection between the arms facing the open arm opposite to the investigator. Each session was video recorded for 5 min and the rat’s position was determined by automatic video tracking (AnyMaze, Stoelting, Co.). The percentage of open arm entries, time in open arms (in seconds, s), time in closed arms (s), and time in the center square (s) was recorded.

### Forced Swim Test

Behavioral despair was assessed using the FST. The rats were placed in a Plexiglas cylinder (20 cm diameter; 50 cm height) filled to a depth of 30 cm with water (23–25°C) for 15 min. The next day, the rats were re-exposed to the swim tanks for a 5 min period. Both swim sessions were video recorded. The time spent immobile was scored by an observer blind to the rat’s housing history. Immobility was defined as a lack of movement but includes the presence of movements necessary to keep the head above water.

### Tissue Preparation and Immunohistochemistry

The day after the last sucrose consumption test, all rats were deeply anesthetized with sodium pentobarbital (340 mg/ml; Euthansol, Merck Animal Health Canada) and then transcardially perfused with room temperature 0.1M phosphate buffered saline (PBS; pH = 7.4) followed by ice-cold 4% (w/v) formaldehyde fixative (pH = 7.4) that was freshly prepared from depolymerized paraformaldehyde. The brains were extracted and post-fixed in the same fixative overnight at 4°C. After fixation, the brains were sectioned on vibrating microtome in the coronal plane at a thickness of 40 μm. All sections were stored at −20°C in a cryoprotectant solution consisting of 30% (w/v) sucrose, 1% (w/v) polyvinylpyrrolidone, and 30% (v/v) ethylene glycol in PBS until use.

To visualize immature neurons, we used the microtubule binding protein doublecortin (DCX) (Brown et al., 2003; Spampanato et al., 2012). DCX immunohistochemistry was conducted as previously described (Fournier et al., 2010) on free-floating sections with all rinses and incubations carried out under gentle agitation. Sections were incubated for 30 min in 0.3% (v/v) H_2_O_2_ to quench endogenous peroxidase. Following a series of PBS rinses, sections then underwent heat-induced epitope retrieval in sodium citrate buffer (pH = 8.5) at 85°C for 30?min and then were placed in a blocking solution containing 0.3% (v/v) Triton X-100, 5% (v/v) normal horse serum and 1% (w/v) bovine serum albumin for 1 h to reduce non-specific antibody binding. The sections were then incubated with rabbit anti-DCX polyclonal antibody (1:1000, Cell Signaling Technology) diluted in blocking solution for 48 h at 4°C. Two days later, the sections were washed several times in PBS and then incubated for 2 h at room temperature with biotinylated goat anti-rabbit IgG secondary antibody (1:500, Vector Laboratories) diluted in 0.3% Triton X-100 in PBS. The sections were then placed in avidin-biotin-peroxidase complex (1:200, 1 h, room temperature, Vectastain ABC Elite, Vector Laboratories) solution and immunolabeling was visualized using 0.02% (w/v) DAB, 2.5% (w/v) nickel ammonium sulfate, 0.083% (v/v) H_2_O_2_ in 0.175M sodium acetate (pH = 7.0) to yield a bluish black product. After sufficient staining, the reaction was halted by washing in PBS several times. The sections were then mounted onto glass slides and left to air-dry overnight. Slides were dehydrated through a series of alcohols, cleared in xylene, and coverslipped with Entellan (Fisher Scientific) mounting medium.

### Doublecortin Stereological Quantification

The total number of DCX + immature dentate granule cells (GCs) was estimated using the unbiased optical fractionator method (West et al., 1991). Every 8^th^ section was examined at 100X (oil immersion) magnification on Nikon Eclipse microscope equipped with a motorized stage and a computerized stereology system (Stereologer). DCX + cells were counted within the dentate granule cell layer and sub-granular zone. The total number of DCX + cells was estimated using the following formula: *N*_total_ = ΣQ^–^^∗^ 1/ssf ^∗^ A(x,y step)/a(frame) ^∗^ t/h; where Σ*Q*^–^ is the number of counted cells; *ssf* is the section sampling fraction (1/8); *A(x,y step)* is the area associated with each x, y movement (90,000 μm^2^) were used to count cells; *a(frame)* is the area of the counting frame (8,019 μm^2^); *t* is the weighted average section thickness; and *h* is the height of the dissector (15 μm). A guard zone height of 2.5 μm on each side of the dissector was used during cell counting to avoid sectioning artifacts. The estimated number of cells represented the total number of cells for the combined left and right dentate gyrus. All cell counts yielded a coefficient of error that was below 0.10.

### Analysis of Maturational Stage of Doublecortin Cells

For analysis of the maturation of DCX + cells, at least 8–10 images of the entire dentate gyrus were captured at 20X magnification using a Nikon TiE inverted research microscope with NIS Elements software. Images acquired were processed with ImageJ. Structural maturation of 50 DCX + cells was evaluated with a staging system based on the classifying cells into one of six stages based on the morphology, length and elaboration of the dendritic tree (Plumpe et al., 2006). Each stage was defined in the following manner: Stage 1: the DCX + cell soma was positioned in the subgranular zone and no dendritic processes were visible. Stage 2: the DCX + cell has 1–2 small short processes that stayed within the subgranular zone. Stage 3: the principal dendrite of the DCX + cell extended into the inner half of the granule cell layer. Stage 4: the leading dendrite reached the outer half of the granule cell layer. Stage 5: the leading dendrite reached the inner molecular layer. Stage 6: the leading dendrite reaches the outer molecular layer. A systematic and random sampling of the DCX + cells was accomplished by placing a 150 × 150 μm grid over the dentate gyrus and only classifying DCX + cells that resided at the points of intersections across the grid. This resulted in total 5–7 DCX + cells per section being examined across the whole hippocampus. The DCX + cells counted for each of the stages was then pooled into early (stages 1 and 2), intermediate (stages 3 and 4) and late (stages 5 and 6) stages.

### Statistical Analyses

Statistical analysis was performed using Statistical Package for the Social Sciences (v. 21.0) statistical software. All data was examined for normality and homogeneity of variance. There were no violations in these parametric assumptions. One-way ANOVA with Fisher’s protected least significant difference *post hoc* tests were used to determine differences between standard house and the LD shifting groups on behavioral test measures as well as DCX + cell counts. Body weight gain, sucrose consumption, and FST data were analyzed using a two-way repeated measure ANOVA with time as the within-subject factor and group as the between-subject factor. One-sample *t*-tests statistics were used to examine whether the discrimination indices calculated for each group was significantly different from chance. All data are presented as mean and standard error of the mean.

## Results

### Effect of Chronic LD Shifting on Body Weight

**FIGURE 2:**
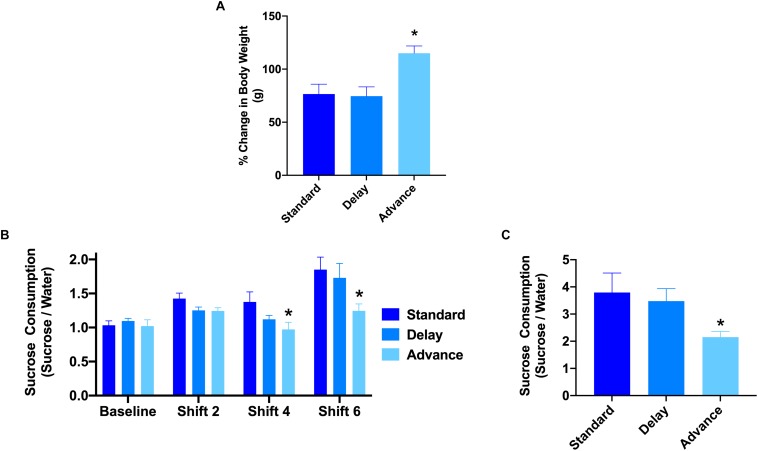
Effect of circadian disruption on body weight and sucrose consumption. **(A)** The proposition of body weight (g) gain (from baseline) in rats housed on a standard, phase advance, or weekly 6-h phase delay LD cycle for 8 weeks. Rats housed on a chronic phase advance schedule weighed significantly more than rats housed on a standard LD cycle rats or rats housed on a chronic phase delay schedule. ^∗^*P* < 0.05. **(B)** Sucrose consumption over a 1 h period was measured before the onset of circadian disruption (baseline) and after cycle shifts 2, 4, and 6. Rats housed on a chronic phase advance schedule consumed significantly less sucrose solution after cycle shift 4 than rats housed on the standard LD cycle. ^∗^*P* < 0.05. **(C)** Sucrose consumption test over a 24 h period was measured after the last LD cycle shift (shift 8). Rats housed on a chronic phase advance schedule consumed significantly less sucrose solution over the 24 h period than rats housed on the standard LD cycle or rats housed on a chronic delay schedule. ^∗^*P* < 0.05.

All rats showed a progressive increase in body weight over the duration of the experiment. A two-way repeated measures ANOVA revealed a significant time by housing condition interaction in the relative change in body weight from baseline to the end of the behavioral testing [*F*(12,126) = 2.69, *P* < 0.003, Figure 2A]. The major source of the interaction was the higher change in body weight at cycles 5, 6, and 8 of the experiment for the rats housed on the phase advance schedule compared to those from the standard and phase delay groups [All *P*s < 0.05]. In addition, when examined over the entire duration of the study, the rats housed on the phase advance schedule weighed significantly more than standard (*P* < 0.046) and phase delay (*P* < 0.030) groups.

### Effect of Chronic LD Shifting on Anhedonia

To assess the potential effects of chronic LD shifts on anhedonia, the rats were presented with a drinking bottle that contained 1.5% sucrose solution for a period of either 1 or 24 h. The results of a repeated measures ANOVA with time (baseline, cycles 2, 4, 6) as the within-subject factor and housing condition (standard vs. advance vs. delay) revealed a significant main effect of group [*F*(2,21) = 5.98, *P* < 0.008]. The major source of the difference was the reduction in sucrose consumption during a 1 h test at cycles 4 and 6 for rats housed on a chronic phase advance schedule compared to rats maintained on a standard LD cycle (Figure 2B). In addition, examination of sucrose consumption over a 24 h period after the completion of behavior testing (i.e., at the end of cycle 8) further confirmed a decrease in sucrose consumption for rats maintained on the phase advance schedule (advance vs. standard: *P* = 0.031, advance vs. delay: *P* = 0.077, Figure 2C). There was no significant difference in general water consumption at any time point during the study (data not shown; All *P*s > 0.149) suggesting that the change in sucrose preference was not related to general alterations in fluid consumption.

### Effect of Chronic LD Shifting on Exploratory Behavior

**FIGURE 3:**
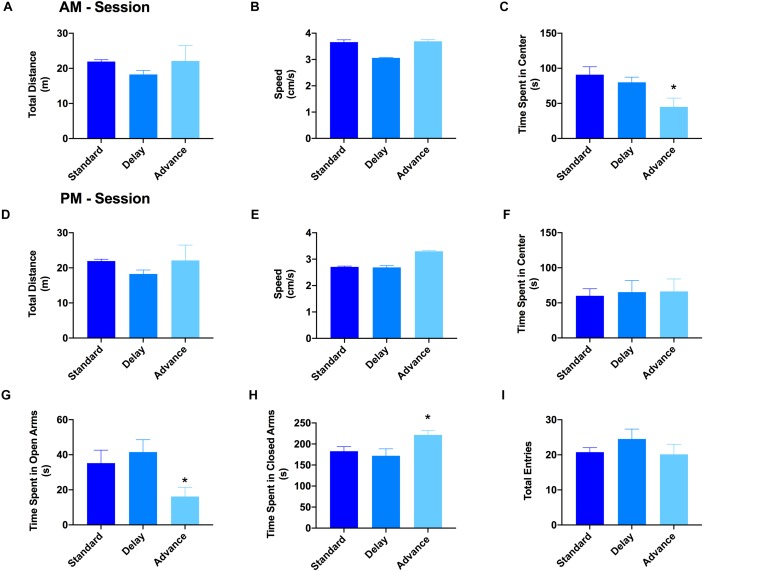
Effect of circadian disruption on exploratory behavior. The total distance traveled **(A)**, movement velocity **(B)**, and time spent in the center **(C)** of the open field during the AM session. There was no difference in either the total distance traveled or movement velocity for rats housed on a weekly phase advance, phase delay or standard LD schedule. However, rats housed on a chronic phase advance schedule traveled and spent significantly less in the center compartment of the open field arena compared to rats housed on the standard LD cycle or rats housed on the phase delay schedule. The total distance traveled **(D)**, movement velocity **(E)**, and time spent in the center **(F)** of the open field during the PM session. There were no differences between any of the groups on these measures. Total time spent in the open **(G)** and closed **(H)** arms of the elevated plus maze. Rats chronically housed on the phase advance schedule spent significantly less time in the open arms of the elevated plus maze. **(I)** Total arm entries (open + closed arms) in the elevated plus maze. There was no significant difference in overall arm entries between groups. ^∗^*P* < 0.05 from standard and phase delay groups.

The open field test and elevated plus maze test were used to evaluate the patterns of explorative behavior and anxiety. For the open field test, the rats were habituated to the open field arena twice (10 min each) on a single day (6 h between exposure). For the first session, the results of a one-way ANOVA showed a significant effect of housing condition on the time spent in the center region of the open field arena [*F*(2,21) = 7.06, *P* < 0.005, Figure 3C]. *Post hoc* analyses revealed that the major source of this difference was the reduced activity of the rats housed on the phase advance schedule in the center region compared to those on the phase delay [All *P*s < 0.001, Figure 3C] or standard LD cycle [All *P*s < 0.024, Figure 3C] groups. There was no difference across the groups with respect to the total distance traveled (Figure 3A) or movement velocity (Figure 3B). Interestingly, movement velocity in the peripheral compartment, but not center compartment, was significantly reduced for the rats housed on the phase advance schedule compared to the two other groups [*F*(2,21) = 5.91, *P* < 0.009]. For the second session, none of the behavioral parameters (e.g., time spent in center compartment, total distance traveled, movement velocity) were significantly different between groups (Figures 3D–F).

The results from the open field arena suggested that the initial reduction of the center compartment during the first session might have been related to increased anxiety or avoidant behaviors induced by the novelty of the environment. To explore this possibility, a second test for anxiety and exploratory behavior was conducted using the elevated plus maze. There was a significant effect of housing condition on the time spent in the open arms of the elevated plus maze [*F*(2,21) = 3.96, *P* < 0.035]. As shown in Figures 3G,H, the rats housed on a chronic phase advance schedule spent significantly less time in the open arms and more time in the closed arms compared to rats maintained on a standard LD cycle [phase advance vs. standard LD controls, *P* < 0.013]. There was no significant effect for housing condition on the total number of arm entries [*F*(2,21) = 0.971, *P* = 0.395, Figure 3I].

### Effect of Chronic LD Shifting on Recognition Memory

**FIGURE 4:**
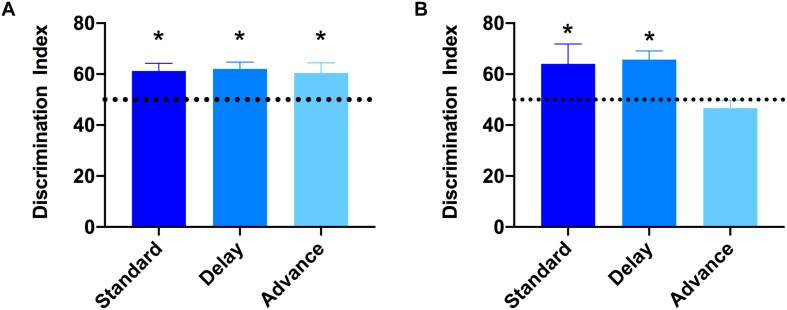
Effect of circadian disruption on object recognition memory. **(A)** Discrimination index for the 15 min retention interval. All groups successfully discriminated the novel object over the familiar object at levels that were greater than 50% chance (^∗^one-sampled *t*-test, *P* < 0.05). **(B)** Discrimination index for the 60 min retention interval. Rats housed on a chronic phase advance schedule did not discriminate the novel object over the familiar object at levels greater than 50% chance. ^∗^*P* < 0.036 from chance for standard and phase delay groups.

To examine if chronic LD shift schedules could impair cognitive function, the novel object recognition test was performed. None of the groups showed preference for either of the two identical objects presented during the sample (acquisition) phases (All *P*s > 0.652). After the 15 min retention interval, a one-sample *t*-test was used to examine whether the discrimination index was significantly different from 50% (chance level). As shown in Figure 4A, all groups showed a comparable level of discrimination for the novel object after the 15 min retention interval [standard: *t*(7) = 2.64, *P*s < 0.033; phase delay: *t*(7) = 4.16, *P* < 0.002; phase advance: *t*(7) = 3.72, *P* < 0.007, Figure 4A]. However, when the retention interval was increased to 60 min, the discrimination index for rats housed on the phase advance schedule did not differ significantly from chance [phase advance: *t*(7) = 1.08, *P* = 0.315, Figure 4B], whereas rats housed on either phase delay schedule or the standard condition all showed a significantly greater preference for the novel object [phase delay: *t*(7) = 2.60, *P* = 0.036; standard: *t*(7) = 4.56, *P* = 0.003, Figure 4B].

### Effect of Chronic LD Shifting on Depressive-Like Behavior

**FIGURE 5:**
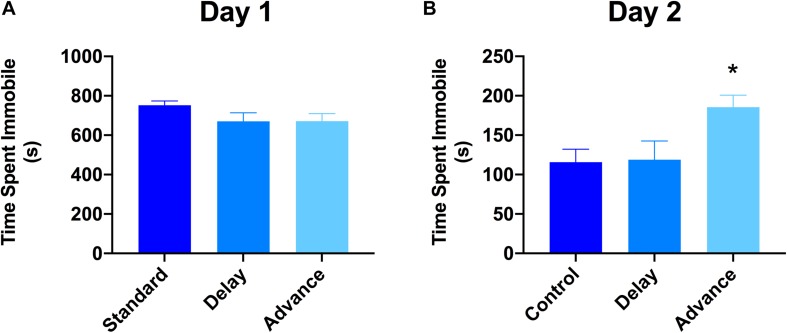
Effect of circadian disruption on FST. **(A)** Total time spent immobile on day 1 of a 15 min FST. **(B)** Total time spent immobile on day 2 of a 5 min FST. Rats that were chronically housed on a weekly phase advance schedule spent significant more time immobile than all other groups on day 2 of the FST. ^∗^*P* < 0.05 from standard and phase delay group.

The 2-day version of the FST was used to examine whether chronic LD shifting increases responsiveness to stress (behavioral despair). The first FST session lasted for 15 min, whereas the second FST lasted for 5 min, and these two sessions were separated by 24 h. The analysis of data on the first day of the FST showed that all groups exhibited similar levels of immobility during this test [*F*(2,21) = 0.223, *P* = 0.802, Figure 5A]. However, for the second day of FST, rats housed on phase advance LD schedule exhibited significantly higher levels of immobility compared to the other groups [*F*(2,21) = 4.39, *P* < 0.026; phase advance vs. standard, *P* < 0.016, phase advance vs. phase delay, *P* < 0.021, Figure 5B]. Further analysis revealed that immobility time increased significantly beginning at the third minute of testing and persisted until the end of the session for the phase advance group [*F*(8,84) = 2.24, *P* < 0.032]. In addition, there was a tendency for rats housed on the phase advance schedule to display shorter latencies to the first episode of immobility on the second day of the FST compared to the other groups [phase advance: 24.72 ± 5.76 s vs. phase delay: 34.9 ± 5.45 s vs. standard: 35.2 ± 9.1 s], however, this difference did not reach statistical significance [*P* = 0.255].

### Effect of Chronic LD Shifting on Hippocampal Neurogenesis

**FIGURE 6:**
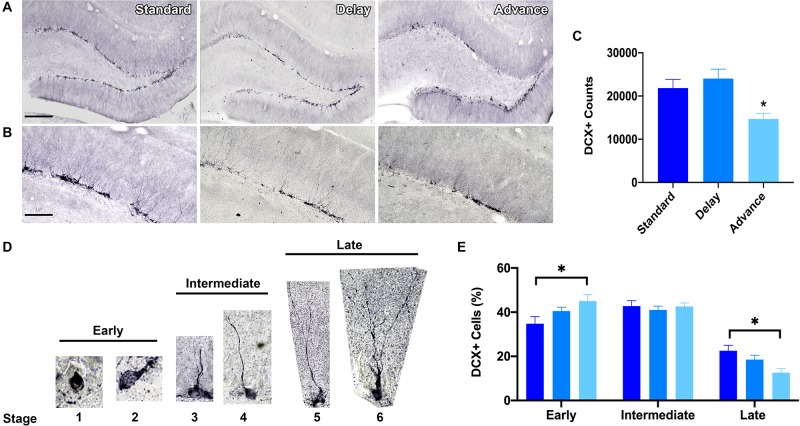
Effect of circadian disruption on hippocampal neurogenesis. **(A)** Representative (10x) photomicrographs of doublecortin (DCX) immunolabeling in the adult dentate gyrus of a rat in each housing condition: standard housing, weekly 6 h phase delay schedule, or weekly 6 h phase advance schedule. Scale bar: 300 μm. **(B)** Representative (20x) photomicrographs of DCX immunolabeling in the adult dentate gyrus of a rats in each housing condition: standard housing, weekly 6 h phase delay schedule, or weekly 6 h phase advance schedule. Scale bar: 100 μm. **(C)** Quantitative stereological estimates at the number of immature DCX + cells in the dentate subgranular zone. Rats housed on a chronic 6 h weekly phase advance schedule had significantly less DCX + cells in the dentate subgranular zone than rats housed on a standard LD cycle, or rats housed on a chronic 6 h weekly phase delay schedule. **(D)** Immature adult born DCX + GCs were classified into three distinct stages according to the degree of their structural maturation (i.e., orientation, outgrowth of dendritic processes in the dentate subgranular zone). DCX + cells were considered to be in the early stage (stage 1 and stage 2) when the soma was positioned in the subgranular zone (SGZ) and no dendritic processes were visible (stage 1) or when the cell displayed short processes that were located within the SGZ (stage 2); DCX + cells were considered to be in the intermediate stage (stages 3 and 4) when the principal dendritic process projected into the inner half of the granule cell layer (stage 3) or when the leading dendrite reached the outer half of the GCL (stage 4); DCX + cells were considered to be in the late stage (stage 5 and stage 6) when the leading dendrite extended into the inner molecular layer (stage 5) or when the leading dendrite reached the outer molecular layer (stage 6). **(E)** Classification of adult-born DCX + cells into early, intermediate and late stages revealed a marked shift in stage distribution. Rats housed on the weekly phase advance schedule had a higher proportion of early stage DCX + cells and a reduction in late stage DCX + cells compared to rats placed on a standard LD cycle or rats housed on a weekly phase delay schedule. ^∗^*P* < 0.05.

To measure the effects of phase shifting schedules on hippocampal neurogenesis, we quantified the number of immature GCs in the dentate gyrus using unbiased stereological procedures. A large number of DCX + cells were located in the sub-granular zone or in the inner one-third region of the dentate granule cell layer (Figures 6A,B). The total number of DCX + cells was decreased in the rats housed on the chronic phase advance schedule compared to the standard housed and phase delay groups [*F*(2,21) = 6.90, *P* < 0.005, Figure 6C].

Next, we employed a classification system to examine the degree of structural maturation of the immature dentate GC neurons (Figure 6D). The DCX + cells were classified according to the orientation and outgrowth of apical dendritic processes in the subgranular zone. As shown in Figure 6E, there was a marked shift in the distribution of DCX + cells as a function of housing condition. Rats that were chronically housed on the phase advance cycle had a greater proportion of early stage DCX + cells (stages 1, 2) then standard housed controls [*F*(2,21) = 3.47, *P* < 0.05; phase advance vs. standard, *P* < 0.016]. The soma of these DCX + cells was positioned in the sub-granular zone and these cells had either no dendritic processes or a very short dendritic process. Interestingly, the rats placed on the phase advance cycle also had a significantly lower proportion of late stage DCX + cells than the standard housed controls [*F*(2,21) = 5.47, *P* < 0.012; phase advance vs. standard, *P* < 0.004]. These DCX + cells have a leading dendrite that reached into the molecular layer and often showed branching. However, this difference did not reach statistical significance for the phase delay group [phase delay vs. phase advance, *P* < 0.062].

## Discussion

Our study confirms that experimental simulation of chronic jet lag in male rats can have a number of important consequences. First, we found that frequent phase advances of the LD cycle disrupted the retention of object recognition memory, but only after an interval of 1 h. Second, we found that rats exposed to the phase advance schedule exhibited signature symptoms of depression observed in animal models, including changes in body weight, anhedonia, altered exploratory behavior in a novel environment, and increased immobility in the FST. Third, we showed that rats exposed to the phase advance schedule have lower levels of hippocampal neurogenesis and reduced dendritic complexity of immature dentate GCs compared to controls. And lastly, while phase advances of the LD cycle were found to be disruptive across all measures examined, phase delays had little to no effect. Taken together, these results confirm and extend past findings (Gibson et al., 2010; Fonken et al., 2012; Kott et al., 2012; Ikeno et al., 2016) that repeated phase advances of the LD cycle disrupt neurogenic processes in the hippocampus and produce clear impairments in memory function as well as increases in anxiety and depressive behavior underscoring the importance of proper circadian alignment in regulating normal affect and cognition.

### Chronic Jet Lag Disrupts Memory

Frequent episodes of circadian disruption have well-documented effects on learning and memory. Cho and colleagues observed spatial learning deficits in a group of flight attendants who had experienced repeated exposure to jet lag from working transmeridian flights for more than 3 years (Cho et al., 2000). Similar findings involving cognitive performance deficits have also been reported in industrial workers who had been exposed to frequent shift work (Rouch et al., 2005; Marquie et al., 2015). In rodents, performance on several behavioral tasks were shown to be impaired after chronic jet lag, including the Morris water maze, radial arm, and active/passive avoidance learning (Devan et al., 2001; Loh et al., 2010; Zelinski et al., 2014). In the present study, we found that repeated phase advances to the LD cycles, which can evoke symptoms of chronic jet lag in rodents (Filipski et al., 2004; Davidson et al., 2006; Craig and Mcdonald, 2008; Gibson et al., 2010; Kott et al., 2012), produced impairments in object recognition memory. This effect was observed only after a 1-h delay interval between the sample and test phases suggesting that chronic phase advances might impair consolidative processes involved in either maintaining or stabilizing new memories. At the cellular level, changes in synaptic efficacy and membrane excitability are thought to be critical events in the formation of new memories (Bliss and Collingridge, 1993), and there have been some suggestion that these neuronal properties can be altered during periods of sleep and by sleep disruption (Prince and Abel, 2013). Indeed, multiple studies have shown that sleep deprivation can alter neuronal excitability as well as reduce the strength of long-term potentiation, a cellular marker of memory, induced in hippocampal slices from sleep-deprived rats (Mcdermott et al., 2003; Marks and Wayner, 2005).

### Chronic Jet Lag Increases Anxiety and Depressive-Like Behaviors

In addition to cognitive effects, evidence supporting an association between circadian rhythm disruptions and changes in mood and anxiety has been slowly accumulating (Bedrosian and Nelson, 2017). Modifications in lighting conditions and aberrant LD cycles can affect mood-like behavior in rodents. For example, anxious- and depressive-like behavior is evident in mice exposed to dim light during nocturnal periods (Bedrosian et al., 2011, 2013; Fonken and Nelson, 2013). Furthermore, short photoperiods are associated with elevated depression in diurnal rodents (Ashkenazy et al., 2009; Ashkenazy-Frolinger et al., 2015) and in some nocturnal rodent species (Prendergast and Nelson, 2005; Prendergast and Kay, 2008; Monje et al., 2011). While these findings clearly show that alterations in day length and light intensity can have an influence on affective states, the impact of chronic LD phase shifts is less clear.

The present study used multiple behavioral measures to examine the impact of chronic phase shifts on depression and anxiety. Anhedonia (i.e., diminished interest or pleasure in all or most activity most of the day) is a core diagnostic symptom of depression and can be readily modeled in rodents using the sucrose consumption test (Scheggi et al., 2018). Reduced sucrose consumption is taken as a measure of anhedonia; an interpretation validated by the demonstration that the same rats do not exhibit reduced consumption to water (Papp et al., 1991; Matthews et al., 1995). Consistent with this idea, we found that rats subjected to weekly phase advances consumed less sucrose than rats housed on a standard LD cycle or those placed on a weekly phase delay schedule. Decreased sucrose consumption was apparent after cycle 4 and persisted at each time point of assessment until the end of the study. Importantly, there was no difference in water consumption between the groups at any phase of the study arguing against the possibility that alterations in sucrose intake reflect a tendency toward reduced thirst. Next, we examined the effect of chronic jet lag on behavioral responses in the FST—a widely utilized rodent model of behavioral despair (Kara et al., 2018; Molendijk and De Kloet, 2019). Rats repeatedly exposed to phase advances engaged in more immobility during the second FST than all other groups. Finally, rats placed on the phase advance schedule also engaged in less time exploring the center portion of a novel open field arena and spent less time in the open arms of the elevated plus maze. Decreased exploration of these regions is widely accepted to reflect higher levels of anxiety behaviors in rodents (Belzung and Griebel, 2001; Walf and Frye, 2007).

Our findings also suggest a specific impact of the direction of phase shifts of the LD cycle on affective behaviors, namely that chronic phase advances induce higher levels of depression and anxiety in rats than phase delays. This is in line with evidence in both human (Aschoff et al., 1975) and rodents (Zee et al., 1992; Gibson et al., 2010; Kott et al., 2012; Zelinski et al., 2014) that phase advances of the LD cycle are typically more disruptive to physiological and behavioral processes than phase delays. Indeed, previous work has shown that reentrainment to a 6 h phase delay in mice occurs within a couple of days, whereas reentrainment to a phase advance takes at least 5 to 6 days to occur (Reddy et al., 2002). Anyan et al. (2017) found that the rate of reentrainment after a 6 h phase advance was associated with higher levels of anxiety behavior in rats, reflected as less time in the center of an activity box and reduced time spent in the open arms of the plus maze, a finding that mirrors our own. While the effect of acute circadian shifts on emotional behavior is clear, research on the impact of chronic jet lag on the development of mood disorder has been limited. However, several studies have shown that acute episodes of jet lag can exacerbate depressive and anxiety symptoms in clinically depressed patients (Tec, 1981; Jauhar and Weller, 1982; Young, 1995; Inder et al., 2016). Our findings are consistent with this observation and further suggest that chronic circadian disruption after repeated phase advancements can impact the function of neural circuits important in mood regulation and anxiety behavior. Finally, the impact of chronic jet lag on female heath has been increasingly recognized (Logan and Mcclung, 2019). While our study only examined the effect of jet lag simulation in male rats, we predict that repeated phase advances of the LD cycle could exert greater effects in females particularly regarding emotional behaviors. Indeed, female flight attendants are 2 to 5.7 times more likely to develop depression and anxiety disorders than the general population (Mcneely et al., 2014). Nonetheless, additional work will be necessary to determine whether sex differences may exist in the behavioral and cellular responses to chronic circadian disruptions that simulate conditions of jet lag.

### Impact of Chronic Jet Lag on Hippocampal Neurogenesis

Although the functional role of adult hippocampal neurogenesis is still unclear, increasing evidence has highlighted an important contribution of ongoing neurogenesis in learning and plasticity, as well in mood and stress reactivity (Cameron and Glover, 2015; Kropff et al., 2015). In rodents, exposure to chronic stress and behavioral models that induce depressive-like behavior are well-known disruptors of hippocampal neurogenesis leading to the idea that impaired neurogenesis might play as a role in the development of depression and other affective disorders (Fournier and Duman, 2012). Given our finding that chronic phase advances of the LD disrupted recognition memory and increased depressive-like behaviors, we considered that chronic phase advances might act as a stressor that could impact levels of hippocampal neurogenesis. In support of this, we found that the total number of DCX + cells was significantly reduced in rats repeatedly exposed to phase advances of the LD cycle compared to those housed on a chronic phase delay cycle or those who remained on a constant 12/12 h LD cycle. These findings are in accordance with the conclusions reached from two independent studies demonstrating that circadian disruptions mimicking jet lag conditions can reduce levels of neurogenesis (Gibson et al., 2010), and that the severity of the neurogenic deficits appear to be direction-dependent with repeated advancements of the LD cycle producing a higher suppression in cell proliferation and neurogenesis than phase delays (Kott et al., 2012).

Because DCX is expressed not only during the initial steps of neuronal differentiation but also during other periods of maturation of young neurons, such as synaptogenesis (Couillard-Despres et al., 2005; Plumpe et al., 2006), we also determined whether chronic jet lag might affect the structural maturation of immature (DCX+) neurons. We found that repeated phase advances increased the proportion of early stage post-mitotic DCX + cells (stages 1 and 2) but decreased the proportion of late stage DCX + cells (stages 5 and 6). Late stage DCX + cells are characterized by the growth of established dendritic processes that extend into the inner and outer molecular layer, and this phase of development is associated with intense spine synapse formation and circuit integration (Zhao et al., 2006, 2015; Toni and Sultan, 2011; Vivar and Van Praag, 2013; Radic et al., 2015; Beining et al., 2017; Petsophonsakul et al., 2017). The potential loss of the later stage DCX + cells, but not early stage DCX cells could suggest that chronic phase advancements might interfere with processes associated with cell maturation and/or survival as we observed a net decrease in the number of DCX + cells in this group. There is evidence that repeated phase advancements increase corticosteroid levels (Gibson et al., 2010) and can potentiate responses of the HPA axis to stress (Loh et al., 2010). Prolonged periods of stress along with aberrant glucocorticoid signaling are well-known to impair the proliferation and structural maturation of neuronal progenitors in the adult hippocampus (Wong and Herbert, 2006; Schoenfeld and Gould, 2013). Similar to our findings, Lussier et al. (2011) observed that chronic high doses of corticosterone in rats for 21 days selectively decreased the number of late phase DCX + cells and reduced dendritic complexity immature (but not mature) granule cells. Thus, it is possible that that elevated levels of GR signaling associated with frequent phase advancements might slow the maturational development of newborn neurons resulting in a decrease in late stage neural progenitors.

## Conclusion

Chronic circadian rhythm misalignment is a core feature of many neuropsychiatric conditions, including mood and anxiety disorders, and there is increasing evidence that disrupted circadian rhythms might be involved in development of these disorders. Our findings demonstrate that chronic phase advancements of the LD cycle, an experimental model of jet lag, produce impairments in object recognition memory and increases depression and anxiety behavior in male rats. In addition, chronic phase advances also decreased levels of hippocampal neurogenesis and appeared to impair the maturation of immature neurons. In sharp contrast, chronic phase delays produced limited effects on behavior as well as levels of hippocampal neurogenesis suggesting the possibility of a direction-dependent effect of chronic jet lag conditions.

While the mechanism that mediates the adverse effect of weekly phase advances observed in this study is unclear, different responses of the circadian system to phase advances and delays have been described. For example, behavioral reentrainment in rodents takes longer after phase advances than phase delays (Reddy et al., 2002), which agrees with observations that circadian clock gene expression is more readily disrupted with phase advances of the LD cycle (Nagano et al., 2003). Indeed, the severity of jet lag symptoms is known to be influenced by the direction of travel and the number of time zones crossed with eastward flights (i.e., phase advances) taking longer to reentrain circadian processes than westward flights (i.e., phase delays) (Waterhouse et al., 2007). Phase shifts can impact glucocorticoid secretion and stress responses, however, these effects tend to be larger and more sustained with repeated phase advances (Gibson et al., 2010; Kiessling et al., 2010; Loh et al., 2010) suggesting that phase advances of the LD cycle may function as a form of chronic stress. Given the observed link between stress and circadian disruption, it is interesting to note that chronic stress can alter molecular clock gene expression in several brain areas known to be critically involved in mood regulation and learning, such as the amygdala, nucleus accumbens, prefrontal cortex, and hippocampus (Savalli et al., 2014; Logan et al., 2015; Landgraf et al., 2016b). Interestingly, hippocampal clock gene expression can regulate the proliferation, maturation, and survival of adult-born neurons (Bouchard-Cannon et al., 2013; Malik et al., 2015) and several lines of evidence suggests that intact levels of hippocampal neurogenesis is critical for healthy mood and cognition (Fournier and Duman, 2012; Cameron and Glover, 2015). Since these behavioral processes could depend on levels of intact neurogenesis, as well as a variety of biochemical and signal transduction processes that occur across multiple brain regions (Bunney and Potkin, 2008; Vadnie and Mcclung, 2017; Snider et al., 2018), we speculate that chronic phase advances might lead to widespread disruption in circadian clock gating mechanisms thereby contributing to impaired memory and increased emotionality. In summary, our findings underscore the importance of recognizing the impact that chronic disruptions in circadian processes can have on brain function and mental health and suggests the possibility that disruptions in levels of hippocampal neurogenesis along with other neuroplastic changes might contribute to functional impairments associated with chronic jet lag.

## Data Availability Statement

The datasets generated for this study are available on request to the corresponding author.

## Ethics Statement

The animal study was reviewed and approved by Trent University Animal Care and Use Committee.

## Author Contributions

NF designed the experiments. EH, TM, HT, and CC performed the experiments. EH, TM, CC, and NF analyzed the data. HL and NF wrote the manuscript.

## Conflict of Interest

The authors declare that the research was conducted in the absence of any commercial or financial relationships that could be construed as a potential conflict of interest.
